# Properties of Skin in Chinese Infants: Developmental Changes in Ceramides and in Protein Secondary Structure of the Stratum Corneum

**DOI:** 10.1155/2017/3594629

**Published:** 2017-10-02

**Authors:** Chao Yuan, Ying Zou, Yao Xueqiu, Kyoko Shima, Yuki Miyauchi, Ayano Naoe, Satoru Naito, Tsutomu Fujimura, Mitsuyuki Hotta, Takashi Kitahara, Xuemin Wang

**Affiliations:** ^1^Shanghai Skin Disease Hospital, Shanghai, China; ^2^Kao (China) Research and Development Centre Co., Ltd, Shanghai, China; ^3^Biological Science Research, Kao Corporation, Ichikai, Haga, Tochigi, Japan; ^4^Analytical Science Research, Kao Corporation, Ichikai, Haga, Tochigi, Japan

## Abstract

The properties of infant skin regarding its structure and stratum corneum (SC) properties during development compared to adult skin have been reported only for a few races and body sites. The aim of this study was to understand the developmental changes of skin properties in Chinese infants, focusing on SC ceramides and protein secondary structure, which are important for skin barrier function. Three body sites with distinct characteristics (cheeks, inner upper arms, and buttocks) were assessed. Sixty pairs of Chinese infants and their mothers were measured for SC hydration, transepidermal water loss, ceramide levels, sebum with an ester bond, and protein secondary structure of superficial SC. Skin hydration decreased with age at all body sites. TEWL was similar between the 2–12- and 13–24-month-old groups but was higher than the adult group at the buttocks and inner upper arms and was equal to the adult group at the cheeks. These differences coincided with differences in protein secondary structure. Ceramide and sebum levels were lower in the infant groups. We conclude that both the SC functions and the components of infant skin are still developing and are not fully adapted as in adult skin at each body site examined.

## 1. Introduction

Many previous studies have reported the properties of infant skin regarding its functionality, skin structure, and stratum corneum (SC) components [1–13]. Previous reports have suggested that infant skin is not completely the same as adult skin.

Developmental changes in the functionality, structure, and components of infant SC have also been well investigated and have suggested that developmental changes occur drastically during the first month of life and that gradual changes then continue during the infant and toddler periods [2, 4, 7, 9, 10]. However, most of those studies investigated limited races (Caucasians and Japanese) and limited body sites (arms or buttocks). There are only a few reports about Chinese infants [6, 13], and there is much less information about SC components, especially ceramide and protein secondary structure, which are important for SC functionality.

Many infants frequently suffer skin problems such as diaper dermatitis, miliaria, contagious skin problems, atopic eczema, and food allergy eczema. To prevent skin problems and ensure healthy growth during their development and life, understanding the properties of infant skin is important.

In this study, we investigated skin functionality and SC components to characterize the properties of infant skin during development at the buttocks, inner upper arms, and cheeks, which are exposed to different environmental conditions (occluded by a diaper, covered by clothing, and exposed to UV, wind, and climate).

## 2. Materials and Methods

### 2.1. Subjects

The protocol was approved by the Ethical Committees of the Shanghai Skin Disease Hospital and the Biological Science Research Laboratory, Kao Corporation (Tochigi, Japan). Subjects with signed informed consent by their parents were recruited.

A total of 63 pairs of Han-Chinese infants aged 2–24 months and their mothers who had healthy skin were screened from 91 pairs of subjects by a dermatologist. Among those, 60 infants and their mothers were assessed at the buttocks, 32 infants and 58 mothers were assessed at the inner upper arms, and 30 infants and 58 mothers were assessed at the cheeks for all parameters. The evaluated population is shown in Table 1. To minimize stress for the infants caused by the measurements, we limited the time allowed for evaluation, so that some infants who were excited and/or crying, or whose body sites were difficult to keep still were excluded from some evaluations.

### 2.2. Study Design

A dermatologist observed the skin conditions of the infants and their mothers a few days before the examination. Measurements were performed in a climate controlled room maintained at 24–26°C and 40–60% relative humidity. Each measurement area was wiped with a wet paper towel to clean the skin surface, after which the skin was acclimatized for 20 minutes. The use of skin care products (e.g., moisturizer, cream, and balm) was prohibited after bathing on the day before the examination. The measurements were performed at the 3 body sites of each infant and mother (buttocks, inner upper arms, and cheeks).

### 2.3. Measurements

Skin hydration (capacitance) was measured using a Corneometer® CM (Courage & Khazaka GmbH, Köln, Germany), and average values from 10 measurements for each body site were calculated. TEWL was measured using a Tewameter® (Courage & Khazaka electronic GmbH, Köln, Germany), and average values from 5 measurements for each body site were calculated. The amount of sebum with an ester bond and the index of protein secondary structure (*β*/*α* defined by the signal ratio of *α*-helix and random coil and *β*-sheet) were measured using a Fourier Transform Infrared (FT-IR) Spectrometer (Flexscan, A2 Technologies, Santa Clara, United States), and average values from more than 3 measurements for each body site were calculated. These parameters were obtained from the infrared spectrum of the skin based on the calculation method previously reported [14, 15].

### 2.4. Analysis of Ceramide Species in the SC

SC specimens were collected from 5 tape-strips from each skin site using a 2.5 × 5 cm square tape (PPS tape, Nichiban, Co., Ltd, Tokyo, Japan). We used half of each piece of tape (2.5 × 2.5 cm) to determine the protein content, and the other half to analyze the ceramide content. Proteins were extracted with a 1% SDS, 0.1 M NaOH aqueous solution, and measured using a BCA kit (Thermo Scientific, Barrington, IL, USA) with bovine serum albumin as a protein standard. After the extraction of ceramides from the tape using a MeOH/chloroform solvent, ceramides were analyzed using normal phase liquid chromatography electrospray ionization mass spectrometry (LC-ESI-MS, Agilent 1100 Series, Agilent Technologies, Palo Alto, CA, USA) as detailed previously [16, 17]. To comprehensively quantify ceramides, including those not available as authentic species, we used a procedure to estimate their levels using relative responses of representative authentic species covering the species targeted as previously reported [16, 17]. The range of relative standard deviation (RSD%) of this analytical method is approximately 10% [17]. Finally, we got ceramide level normalized by protein content.

### 2.5. Statistical Analysis

Significant differences of each parameter and component among the 3 groups (2–12-month-old infants, 13–24-month-old infants, and their mothers) were tested with the Bonferroni-test.

## 3. Results

### 3.1. Skin Hydration and Transepidermal Water Loss (TEWL)

Skin hydration (capacitance) and TEWL measurements were performed using a Corneometer and a Tewameter, respectively.  Table 2 shows the results at the buttocks, the inner upper arms, and the cheeks. The data are grouped by age (2–12 months old, 13–24 months old, and mothers). We observed common alterations at all three body sites. The water content decreased in an age-dependent manner. In the 13–24-month-old group, a higher water content than the mothers was observed at the buttocks, while a significantly lower water content than the mothers was observed at the inner upper arms and cheeks.

TEWL at the buttocks and inner upper arms was significantly higher in both age groups of infants compared to the mothers. In contrast, no significant difference was found in TEWL at the cheeks between infants in both age groups and the mothers. There was no significant difference between the 2 age groups of infants at any body site.

### 3.2. Ceramide in the SC

**Figure 1:**
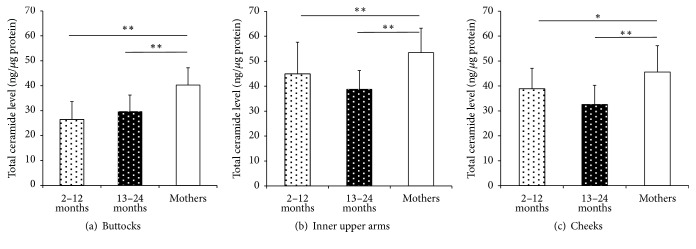
Total ceramide level of the buttocks, inner upper arms, and cheeks. (a) Total ceramide level at the buttocks for each age group (2–12-month-old (2–12 M): *n* = 28; 13–24-month-old (13–24 M): *n* = 32; mothers: *n* = 60) (^*∗∗*^*p* < 0.01). (b) Total ceramide level at the inner upper arms for each age group (2–12 M: *n* = 17; 13–24 M: *n* = 15; mothers: *n* = 58) (^*∗∗*^*p* < 0.01). (c) Total ceramide level at the cheeks for each age group (2–12 M: *n* = 14; 13–24 M: *n* = 16; mothers: *n* = 58) (^*∗*^*p* < 0.05, ^*∗∗*^*p* < 0.01). Data are shown as means ± SD.

Figure 1 shows the total ceramide level at the buttocks (a), inner upper arms (b), and cheeks (c). The total ceramide level was significantly lower in infants in both age groups than in mothers at all body sites. There was no significant difference between the 2–12-month-old group and the 13–24-month-old group at any skin site.

### 3.3. Superficial Components of the SC Determined by FT-IR Spectroscopy

**Figure 2:**
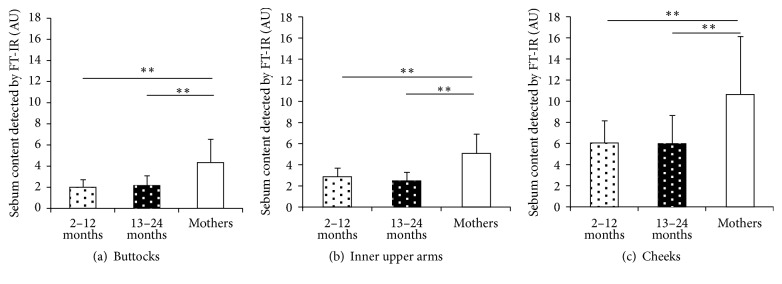
Sebum with an ester bond content at the buttocks, inner upper arms, and cheeks. (a) Sebum with an ester bond level at the buttocks for each age group (2–12-month-old (2–12 M): *n* = 28; 13–24-month-old (13–24 M): *n* = 32; mothers: *n* = 60) (^*∗∗*^*p* < 0.01). (b) Sebum with an ester bond level at the inner upper arms for each age group (2–12 M: *n* = 17; 13–24 M: *n* = 15; mothers: *n* = 58) (^*∗∗*^*p* < 0.01). (c) Sebum with an ester bond level at the cheeks for each age group (2–12 M: *n* = 14; 13–24 M: *n* = 16; mothers: *n* = 58) (^*∗∗*^*p* < 0.01). Data are shown as means ± SD.

Superficial components of the SC that existed at the first 1-2 microns of the SC were characterized using FT-IR. Sebum has characteristic IR signal of an ester bond at 1740 cm^−1^, and protein has that of Amide I at 1650 cm^−1^. Thus we can estimate sebum level of SC surface using an ester signal normalized by Amide I.  Figure 2 shows the ester bond level normalized by Amide I signal which is defined as 100.

No significant differences were found between the 2–12-month-old group and the 13–24-month-old group in any of the superficial components of the SC (sebum with an ester bond, ratio of protein secondary structure). Superficial sebum with an ester bond in both age groups of infants was significantly lower than the mothers for all 3 body sites (Figure 2).

Because the IR signal of Amide I at 1650 cm^−1^ is composed of overlapped multiple IR signals which correspond to the protein secondary structure, we can estimate the protein secondary structure content by analyzing Amide I band shape. We separated Amide I band using the procedure previously reported [14] and calculated the signal ratio of beta-sheet and alpha-helix.

**Figure 3:**
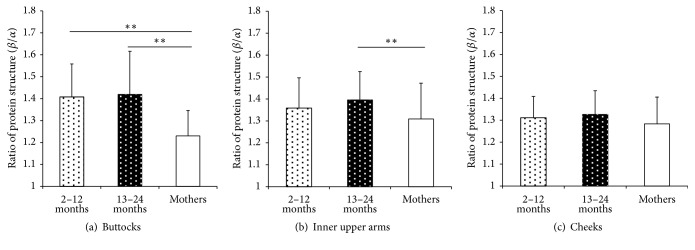
Ratio of protein secondary structure (*β*/*α*) at the buttocks, inner upper arms, and cheeks. (a) Ratio of protein secondary structure (*β*/*α*) at the buttocks for each age group (2–12-month-old (2–12 M): *n* = 28; 13–24-month-old (13–24 M): *n* = 32; mothers: *n* = 60) (^*∗∗*^*p* < 0.01). (b) Ratio of protein secondary structure (*β*/*α*) at the inner upper arms for each age group (2–12 M: *n* = 17; 13–24 M: *n* = 15; mothers: *n* = 58). (c) Ratio of protein secondary structure (*β*/*α*) at the cheeks for each age group (2–12 M: *n* = 14; 13–24 M: *n* = 16; mothers: *n* = 58) (^*∗∗*^*p* < 0.01). Data are shown as means ± SD.

The ratio of protein secondary structure of the superficial SC was significantly higher than the mothers both at the buttocks and the inner upper arms, while no significant difference was found at the cheeks (Figure 3).

## 4. Discussion

There are few published reports about the skin of Chinese infants [6, 13]. One recent investigation reported about 3–49-month-old Chinese infants who lived in Beijing, China [6]. They reported that skin conductance and TEWL decreased with age but were still higher than adults, the same as reported for other countries and races.

Our investigation was designed to elucidate the developmental changes of skin properties in Chinese infants at 3 different environmentally exposed body sites. We evaluated skin functionality and SC components of 2–24-month-old Chinese infants and their mothers at 3 distinct body sites.

We found that SC functionality was still under development until at least 2 years of age (determined by the reduction of capacitance with age at each site, and a higher TEWL than the mothers at the buttocks and inner upper arms) as previously reported [2, 3, 6]. We also found that SC components were not fully acquired like adults, determined by a lower ceramide level, contents of sebum with an ester bond at each site, and the higher protein secondary structure (ratio of *β*/*α*) at the buttocks and inner upper arms.

To understand SC functionality, we analyzed sebum with an ester bond and protein secondary structure (ratio of *β*/*α*) using FT-IR, and SC ceramide using LC-ESI-MS. It is well known that sebum excretion increases in the first week after birth, then becomes lower [10], and our results show that levels of sebum with an ester bond of infants were significantly lower than adults as previously reported [4, 10] and this might be an important reason for the acceleration of water desorption and the reduction of water-holding capacity.

Ceramide is one of the key components that form SC lipid lamellae [18]. Only a few reports have been published about infant skin (epidermal or SC) ceramides [4, 11, 19], and there is much less knowledge about SC ceramides [4]. The ceramide level of infants in this study was significantly lower than the mothers, which is inconsistent with a previous report that evaluated Japanese infants [4]. Hoeger et al. observed that the total epidermal ceramide level of infants increases from the prenatal period to the postnatal period (6 years) [11]. Tachi and Iwamori reported that the epidermal ceramide level of 6-week-old neonates is the same as 20–28 gestational age, while their epidermal ceramide level is lower than adults [19]. As Hoeger et al. noted [11], a lower level of ceramide might mean that the epidermis is still under adaptive responses to the external environment and has not fully adapted to the external environment during the infantile period. Our results of the higher TEWL value at the buttocks and inner upper arms in infants might be related to the lower level of total ceramide. While no similar relationship was observed at the cheeks, the reason for this difference might be related to the higher TEWL value at the cheeks of the mothers compared with the other body sites. We can partially but not completely explain infant SC function by only the ceramide content. The microvascular development, the thickness of the SC, changes in the immune system, and the skin resident flora are all important for exposed skin barrier function.

Keratins are major components of SC protein, and that family has many member molecules, including K1, K2, K5, K10, K14, and K16. It is well known that these keratin molecules reflect the state of keratinization. Each keratin molecule consists of 3 domains, an *α*-helical central rod domain, a nonhelical N-terminal head domain, and a nonhelical C-terminal tail domain. Thus, the ratio of the *α*-helical domain in the SC will depend on its keratin components [20].

In the case of psoriatic skin, the skin is characterized as having decreased levels of K1 and K10 and increased expression of K6 and K16 in the epidermis [21]. Takahashi et al. reported that the SC barrier functional deficiency in psoriatic skin is accompanied by a change of SC protein secondary structure (ratio of *β*-sheet/*α*-helix and random coil) measured using FT-IR [15].

From our observations, although no significant difference was found between the 2–12-month-old group and the 13–24-month-old group, the *β*/*α* ratio was higher in infants than their mothers at the buttocks and inner upper arms, which coincides with the higher TEWL value in infants. Considering the report of the lower content of K1, K10, and K11 and the higher ratio of K6 to K1, K10, and K11 in full-term neonatal SC (at the forehead) compared with adults [22], the higher ratio of *β*/*α* in infant skin suggests that the regulation of keratinization during the infantile period might be different from that of adults.

On the other hand, at the cheeks, no significant difference in the *β*/*α* ratio was found between infants and their mothers, so it seems possible that the protein secondary structure detected by FT-IR was already developed as an adult-like structure by at least 12 months of age at the cheeks. Age-related morphometric changes of inner skin structure in a sun-exposed (cheek) skin site differed from a sun-protected site (arm) [23]. This suggests that exposure to the environment (sun and air) accelerates the development of the skin to adapt to the environment.

*Limitations*. In this study, regarding differences between younger and older infants, we observed age-related alterations only in SC hydration, but no other alterations were observed in other parameters. One reason might be that the testing sample size was not big enough, but another important reason was suggested from a previous report [2, 4, 9] that drastic structural and functional alterations occur during the first month of life and then occur gradually after that.

## 5. Conclusion

To understand developmental alterations of infant skin, we evaluated ceramide levels by the tape-stripping method and components of superficial SC using a noninvasive method. From our observations, a lower level of ceramide was observed in 2–24-month-old infants than in the adult group, which suggests that the development of lipid metabolism is not yet fully adapted as in adults. The ratio of protein secondary structure (the infrared signal ratio of *β*-sheet/*α*-helix and random coil) was higher in infants than in adults, and the higher ratio of protein secondary structure in infant skin coincides with a higher TEWL value. The ratio of protein secondary structure might be involved in SC barrier maturation. Determination of the correlation between protein secondary structure and SC components will be needed to further understand the results. However, we have to mention that we could not completely determine SC functionality by these components. To determine SC functionality, expansion of our perspectives to other factors (e.g., the thickness and structure of the skin, the microvascular plexus, microflora, and other components such as proteins and NMF) will be needed. We believe that this fundamental knowledge will be helpful to create a protective approach against skin problems suffered by many infants.

## Figures and Tables

**Table 1:** The evaluated population in each group at three body sites.

	Buttock	Inner upper arm	Cheek
Infants 2–12 months	28	17	14
Infants 13–24 months	32	15	16
Mothers	60	58	58

**Table 2:** Skin hydration (capacitance) and transepidermal water loss (TEWL) of the buttocks, inner upper arms, and cheeks.

	Buttock	Inner upper arm	Cheek
*Capacitance (AU)*			
Infants 2–12 months	55.0 ± 12.1 (*n* = 28)^*∗∗*^	49.1 ± 11.5 (*n* = 17)	53.4 ± 9.7 (*n* = 14)
Infants 13–24 months	45.6 ± 11.7 (*n* = 32)^†^	40.8 ± 13.1 (*n* = 15)^*∗*^	39.0 ± 13.4 (*n* = 16)^*∗∗*†^
Mothers	42.5 ± 9.8 (*n* = 60)	47.9 ± 10.3 (*n* = 58)	54.5 ± 16.3 (*n* = 58)

*TEWL (g/m* ^*2*^ *·hr)*			
Infants 2–12 months	23.7 ± 11.8 (*n* = 28)^*∗∗*^	23.2 ± 10.4 (*n* = 17)^*∗∗*^	20.5 ± 8.8 (*n* = 14)
Infants 13–24 months	22.1 ± 9.8 (*n* = 32)^*∗∗*^	26.5 ± 12.4 (*n* = 15)^*∗∗*^	19.0 ± 3.7 (*n* = 16)
Mothers	15.2 ± 7.9 (*n* = 60)	11.5 ± 2.8 (*n* = 58)	19.8 ± 5.5 (*n* = 58)

The Bonferroni-test was used for statistical analyses. Significant difference between the mothers group and the 2–12- and/or the 13–24- month-old infant group: ^*∗*^*p* < 0.05; ^*∗∗*^*p* < 0.01. Significant difference between the 2–12-month-old group and the 13–24-month-old group: ^†^*p* < 0.05.
